# Well-prepared outpatient visits satisfy patient and physican

**DOI:** 10.1136/bmjoq-2018-000496

**Published:** 2019-08-24

**Authors:** Marian Smeulers, Myrte Dikmans, Michèle van Vugt

**Affiliations:** 1 Division of Outpatient Department, Amsterdam UMC-AMC Campus, Amsterdam, The Netherlands; 2 Division of Internal Medicine, Department of Infectious Disease, Center for Tropical Medicine & Travel Medicine, Amsterdam UMC-AMC Campus, Amsterdam, The Netherlands

**Keywords:** quality improvement, ambulatory care, patient satisfaction, electronic patient record, hospital care

## Problem

The increasing number of complex patients, multidisciplinary treatments and mandatory registrations causes an increased administrative burden on medical professionals.^1^ Recent research has shown that for each hour of direct patient care, a physician spends 2 hours on documentation in the electronic patient records (EPRs).^2–5^ This increased administrative burden limits the amount of time and attention of the physician for the patient, as well as the active participation of patients during the outpatient visit.^3 4^

More time can be spent on direct patient contact if the administrative burden during the outpatient visit is reduced, ultimately resulting in better quality of care and increased satisfaction for both patient and physician.

## Situation

Approximately 60 000 new referred patients annually visit the outpatient department of our tertiary care hospital. The hospital introduced a new EPR in 2016, which greatly increased the administrative burden for physicians. This resulted in incomplete medical files that lacked mandatory data according to our hospital policy. One outpatient clinic developed a successful initiative in which physician aides completed parts of the EPR by means of a telephone conversation before the first visit. The positive experiences with this initiative led the divisional board of the outpatient clinics to introduce this method to all outpatient clinics. They commissioned the project ‘Well Prepared Is Half Done’, which was funded with an internal hospital fund. The aim of the project was to complete a basic set of medical data before the patient’s first visit, resulting in a reduction of the administrative burden for the physician.

## Strategy

The project started in 2017 and used a plan, do, study, act (PDSA) approach to adapt and refine an initial dataset, process and telephone script (online supplementary data). The process applies to each new referred patient and consists of a two-step process 1–2 weeks before the visit: step 1, gathering the available data from the referral and/or EPR, and step 2, a telephone call to the patient to obtain, verify and register the data. The conversation ends with the question whether the patient has any questions about the upcoming visit.

10.1136/bmjoq-2018-000496.supp1 Supplementary data

We employed a pool of premedical interns to prepare the outpatient visit. These premedical interns are dedicated to the task, are capable with medical data and are eager to engage with patients. The PDSA cycles were led by a project manager who was responsible for

- Coordination with leadership of the specialisms.
- Refinement of the procedure and dataset based on the feedback of the specialism.
- Management and on-the-job training of the premedical interns.
- Development of the management reports on the completeness of the medical file.

Implementation started with two initial pilot specialisms and a gradual roll-out to other specialisms, 24 in total. Leadership of each specialism was involved to endorse implementation and to instruct the physicians to review and verify the preregistered dataset.

Experiences with preparation of the outpatient visit were evaluated through (1) telephone interviews with patients (a random selection of patients, nine closed ‘yes/no’ questions and a rating score) and (2) questionnaires for physicians (two questions on experienced benefits, a satisfaction score and an open question for further improvement suggestions).

The process of visit preparation was improved through monitoring and evaluation rounds with management of the specialisms. In these meetings, we discussed

- The results of visit preparation (percentage of complete files before the first visit and the time required for file preparation).
- Experiences of the physicians with file preparation.
- Appropriateness of the registered data.
- Feasibility of transferring file preparation to the physician aids.

## Results

### Completeness of medical file

**Figure 1:**
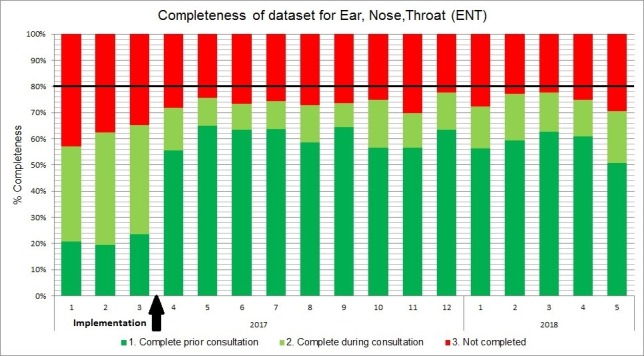
Completeness of dataset before and after first visit preparation for ear, nose and throat.

For each specialism, the medical file completeness was monitored in a chart (figure 1). According to our hospital policy, the target was 80%–100% medical file completeness after the first visit. After implementation, on average, 70% of the patients had a complete dataset before the first visit; 30% of the patients could not be reached; and there was a slight increase of patients with a complete dataset after the first visit. We saw a clear task switching from the physicians to the premedical interns (complete medical file before the first visit).

### Required effort

Overall, the average time to prepare the visit was 14 min per patient. Approximately, 68% of the patients were reached with the first phone call, a second attempt added 24%, and a third/fourth attempt added 8%.

### Satisfaction (patient and physician)

Patients (n=43) awarded the telephone call with an 8.5 out of 10.0. All but one patient experienced the preparation as customer friendly and as a sign of commitment of the hospital to their treatment; they understood the relevance of the questions and they felt heard. Almost all patients appreciated the contact before the visit, and half of the patients also valued an online approach for the questions.

Of the physicians (n=33), 88% experienced benefits from the preparation: they experienced less administrative burden (85%) and a better course of the visit (27%). An improvement suggestion was to instruct the premedical interns on the terminology used for the registration and to tailor and focus the relevant medical history to the specialism.

### Monitoring and evaluation cycle

Overall, management of the specialisms was enthusiastic and keen to continue with the preparation process. Many specialisms had a preference for focused data collection, for example, on the medical history or certain medications. Based on this input, the preparation process was adapted to fit the needs of each specialism.

Most specialisms had doubts about the feasibility of assigning the task of visit preparation to the physician aids. These doubts arose from concerns about the required level of knowledge for the task and whether physician aids had sufficient time to guarantee continuity.

## Discussion

The preparation of the outpatient visit is well received by both patients and physicians and is of added value to both. The personal approach of a telephone call enables tailoring of the questions to the specific situation of the patient. Even though patients indicate that an electronic questionnaire is also an option, this method of querying limits possibilities to tailor the questions and to ask further for clarification; this is especially true for patients with a complex medical history. On the other hand, electronic questionnaires are cheaper to deploy; therefore, it is worthwhile to find out in which situation or for which type of patients an electronic questionnaire is an alternative. Adding electronic questionnaires, combined with the possibility to fill in the electronic questionnaire in the waiting area before the visit, may also contribute to a higher percentage of file completeness, since 30% of patients are not reached by telephone. This way, the intervention for preparing the outpatient visit can evolve into a tailored multifaceted approach.

The pool of premedical interns conducted the preparation with high quality and continuity. A major challenge for the next phase is sustainable implementation of the process in the standing organisation when the task is transferred from the premedical interns to the physician aids. This is also a limitation of our approach because the physician aides have not been involved in the development of this new task that now needs to be embedded in their current tasks. On the other hand, it can enrich their work, and combining file preparation with scheduling the appointment makes the process more efficient.

## Conclusion

This study demonstrated that preparing the outpatient visit by telephone resulted in a reduction of administrative burden for physicians, was well received by patients and was of added value to both. Telephone contact with the patient enables a personalised approach where questions can be tailored to the specific situation of the patient and which also offers the patient the opportunity to ask questions.
