# Supplementary material for: Well-prepared outpatient visits satisfy patient and physican
Source: BMJ Open Qual. 2019 Aug 24;8(3):e000496. doi: 10.1136/bmjoq-2018-000496 (PMC6711440; doi:10.1136/bmjoq-2018-000496)
Supplement: Supplementary data [file bmjoq-2018-000496supp001.pdf]

Supplementary file 1. Dataset and Telephone script

**Introduction**
Introduction, information and agreement from the patient for the call.
Verifcation of the identy of the patient (name + date of birth)
**Administrative data**
Family name, given name, gender
Adress
Email
General practitioner
Other healthcare providers involved in care for the patient
Pharmacy
Reason for referral / main complaint
Allergies/Conta-indications
(Relevant) Medical history
Family anamnesis
Current medication use
Intoxications (alcohol, smoking, drugs), including the amount of usage
First language
Need for interpretator
**Risk screening**
Length and Weight
Unintended weight loss
Screening pain
Screening fall risk
Screening infection risk
Immunization status (children)
**Complete conversation**
Questions the patient might have
Reminder of the date/time of the first visit
